# International Empirical Validation and Value Added of the Multilevel Job Content Questionnaire (JCQ) 2.0

**DOI:** 10.3390/ijerph22040492

**Published:** 2025-03-25

**Authors:** Maren Formazin, Maureen F. Dollard, BongKyoo Choi, Jian Li, Wilfred Agbenyikey, Sung-il Cho, Irene Houtman, Robert Karasek

**Affiliations:** 1 Division “Work and Health”, Federal Institute for Occupational Safety and Health (BAuA), 10317 Berlin, Germany; 2 PSC Global Observatory, University of South Australia, Adelaide 5000, Australia; 3 Center for Work and Health Research, Irvine, CA 92620, USA; 4 Department of Medicine, University of California, Irvine, CA 92617, USA; 5 Department of Environmental Health Sciences, Fielding School of Public Health and School of Nursing, University of California, Los Angeles, CA 90095, USA; 6 US Department of Health and Human Services, Center for Medicare and Medicaid Services, Baltimore, MD 21244, USA; 7 Martin Luther Health Training School, Kintampo Campus, Kintampo, Ghana; 8 Department of Public Health Science, School of Public Health and Institute of Health and Environment, Seoul National University, Seoul 08826, Republic of Korea; persontime@hotmail.com; 9 TNO Netherlands Organisation for Applied Scientific Research, 2333 BE Leiden, The Netherlands; 10 Institute for Psychology, Copenhagen University, 1353 Copenhagen, Denmark; 11 Department of Work Environment, University of Massachusetts, Lowell, MA 01854, USA; 12 Øresund Synergy, Copenhagen, Denmark

**Keywords:** demand, control, stability-support, added variance, JCQ 2.0

## Abstract

This paper investigates whether the Job Content Questionnaire (JCQ) 2.0 composite scales for demand, control, and stability-support at the task and organizational level are related to health and work-related outcomes as hypothesized in the job demand–control and Associationalist Demand–Control models. Further, the relative improvement of the JCQ 2.0 instrument over the JCQ 1 scales in the prediction of health and work-related outcomes is tested. The JCQ 2.0 was applied among workers in Australia and Germany. Analyses of variance and Kruskal–Wallis tests were applied for mean score comparison. In addition, path modeling as well as regression analyses were used. JCQ 2.0 task and organizational level demand, control, and stability-support as well as job strain and organizational-level active work are related to health and work-related outcomes as expected. Associations with active work at the task level are limited. A multilevel framework whereby organizational demands relate to task demands and, in turn, depression and burnout, is found in both German and Australian data. A similar organization to task process is found for control and support in German data, but for Australia, there is only a direct organizational effect on both outcomes. The task- and organizational-level composites—demand, control, and stability-support—explain unique variances in health and work-related outcomes. The JCQ 2.0 composites explain substantially more variance in all outcomes than the classic JCQ 1 DC and DCS scales. The results underline the utility of the JCQ 2.0 to assess multilevel aspects of the psychosocial work environment with broad practical value as a psychosocial risk assessment tool.

## 1. Introduction

Significant evidence has accumulated on the association of psychosocial working conditions using the Demand-Control-Support model (DCS) with both health outcomes and positive behavioral outcomes. Much of the evidence is based on measures—namely, task-level demands, control, and support—derived from the Job Content Questionnaire (JCQ 1). Recently published extensions of both the theoretical base, i.e., the Associationalist Demand–Control (ADC) model (see Appendix A, A.1), and of the corresponding original JCQ to the JCQ 2.0 (see Appendix A, A.2), suggest that it is important to test whether the previously observed “foundational associations” of the DCS model remain valid when using the revised JCQ 2.0. The JCQ 2.0 substantially updates the instrument, shortening previous scales and adding a considerable number of new scales at multiple levels: task, organization, and external-to-work.

Past research has led to extensive and differentiated literature on the associations between job strain and chronic diseases such as cardiovascular diseases (CVD) [1,2,3,4] as well as the onset of musculoskeletal disorders [5] and depressive symptoms [6,7,8]. Likewise, task demands, control, and support are associated with favorable work outcomes [9], such as job satisfaction (JS) [10] and active behaviors outside the workplace [11,12,13].

In this paper, we test the implications of the new JCQ 2.0 instrument, examining the associations between the DCS constructs at the task and the organizational levels and the classic demand–control DC hypotheses predicting workplace outcomes, both negative (health deficit, disease) and positive (active, motivational, learning-related), in an integrated manner. Additionally, we test the combinations of demand and control (job strain, i.e., the combination of high demands and low control, and active work, i.e., the combination of high demands and high control) as the DC approach has done before.

One of the important goals for the JCQ 2.0, as proposed by Karasek and colleagues (see Appendix A, A.1), was to maintain—to the extent possible—the pragmatically and intellectually useful DC narrative. A meta-analysis of 106 studies [14] clearly demonstrates the utility of the Demand-Control-Support framework, as is confirmed by further work [9]. Thus, empirically, the first step was to assess the degree to which factors for demand, control, and support at the task and organizational level, based on numerous sub-scales, namely the JCQ 2.0 scales, function as previously hypothesized. Analysis of the structure of the JCQ 2.0 (see Appendix A, A.3) found sufficient coherence across four pilot studies in four countries and across the two levels to ensure that the DCS constructs are empirically usable at multiple levels, allowing us to apply composite scores for further analyses.

Thus, in this paper, we combine scale scores belonging to the same factors (see Appendix A, A.3) and analyze the predictive validity of these six composite scores for health and work-related outcomes. The use of such composite scales will substantially simplify testing, presenting, and understanding of the findings—given four pilot studies, two testing levels, up to seventeen scales (in later pilot versions), and six dependent variables. However, analyses of composite scores may overlook important associations for many of the individual JCQ 2.0 scales (see Appendix A, A.2) that are further examined in an additional paper (see Appendix A, A.4). We note that using the composite scores—summing over the component scales equally weighted—is not the same as using, for example, the underlying factor scores from a structural equation model.

In this paper, we conduct three different forms of dependent measure tests:

1. We use the JCQ 2.0 scales, in composite form, to test the association of the JCQ 2.0 dimensions with dependent variables, including worker health and favorable work outcomes.
2. We review the relative strength of association for JCQ 2.0 composite task and organization level scales with two dependent variables using a simplified path model analysis.
3. We test the relative improvement that the JCQ 2.0 instrument provides in “predicting” (albeit cross-sectionally) associations over the base JCQ 1 items in both Australia and Germany.

With these tests, we hence aim at showing that (i) the JCQ 2.0 composite scales for demand, control, and stability-support at both the task and the organizational level are related to health and work-related outcomes as hypothesized in the DC and ADC models, and (ii) the JCQ 2.0 explains more variance in health and work-related outcomes than the scales solely from the JCQ 1.

It should be noted that the organizational-level scales only exist in a full, practically relevant set in the two later pilots, Australia and Germany. However, a limited comparative review of task-level associations is provided for Korea and China with regard to the first analyses.

### 1.1. New Theoretic Base

The new systems-theoretic base for the multilevel demand, control, and stability-support (DCS-S) concept is the ADC theory (see Appendix A, A.1). The basic building blocks of DCS are here translated to fit a new multilevel framework, which specifies expanded and refined DCS constructs at the task level and new DCS-S elaborations at the organizational level. Karasek et al. (see Appendix A, A.1) describe how the JCQ 2.0 differs from its predecessor, the JCQ 1, in capturing organizational-level working conditions and how these impact working conditions at the task level. Specifically, they describe the expansion of support at the task level to stability-support at the organizational level (see Appendix A, A.1). The ADC model involves workers and organizations (a) doing their daily “work” as necessary for their adaptation to their environment and then (b) building “ordering capacity” to restore their capability for further actions necessary for continual existence of healthy wellbeing and growth. The original DC model’s dual hypotheses about workers’ health and wellbeing reflect “energy and order” related working conditions. Utilization of these very general and abstract “energy and order” themes allows for building DC narrative-consistent, broadly generalizable extensions in a multilevel JCQ 2.0 workplace social context. While the JCQ 1 focused on task implications, the focal theoretical innovations for the JCQ 2 represent extensions of “control” as multilevel work-related action pathways. The expanded multilevel demands concept reflects both the worker’s possibility of maintaining a personal equilibrium in daily working life and at the same time the organization’s need to continually bring in new resources of effective adaption and further growth. The dynamic nature of this balance of challenges is reflected in the organizational mid-level concept of Platforms of Dynamic Stability as discussed by Karasek et al. (see Appendix A, A.1). Overall, ADC theory describes how systems can either organize themselves into higher levels of complexity (extending the Active Work hypothesis and “health” as in the established DC model)—i.e., systems that grow and develop—or, alternatively, how complex systems devolve to lower levels of complexity (extending the Job Strain hypothesis of the DC model), no longer able to sustain their original capability. While empirical testing of the full range of ADC theory’s dynamic hypotheses is beyond the capacities of the current cross-sectional datasets, the test below can verify the new instrument’s capability of meaningfully extending the original DC narrative in our current global economic context and is thus both a feasible and a necessary requirement for meaningful empirical testing of the JCQ 2.0.

### 1.2. JCQ 2.0 Task-Level Constructs and Associations

Given the modifications to the JCQ 2.0 tool compared to the JCQ 1 [15], shortening the available scales and adding new scales, it is important to verify the foundational associations of the DCS model using the revised JCQ 2.0 tool. We propose:

**Hypothesis** **1.** High task-level demands will be positively related to poor health (poor self-rated health (SRH), burnout, depression) and negatively related to favorable work outcomes (affective commitment to the organization (AC)/work engagement (WE), JS, and intention to stay (ITS)/intention to leave (ITL)).

**Hypothesis** **2.** High task-level control and support will be negatively related to poor health and positively associated with favorable work outcomes.

It is important to also verify, using the new tool, the foundational strain and active dimensions specified in the DC model at the task level and their associations with health and favorable work outcomes, respectively, that have been found in previous research [16,17,18]. For the revised JCQ 2.0, we propose:

**Hypothesis** **3.** Workers with high job strain at the task level have higher levels of poor health than workers with low job strain.

**Hypothesis** **4.** Workers with high task-level active work have higher levels of favorable work outcomes than workers with passive jobs [12].

### 1.3. JCQ 2.0 Organizational-Level Constructs and Associations

Moving to the organizational level framework, the separate components are organizational demands, control, and stability-support. Demands are conceived in terms of organizational disorder and restructuring, assessing organizational processes and change affecting job demands for employees, respectively; for more detailed definitions of all JCQ 2.0 measures, please refer to Karasek et al. (see Appendix A, A.1).

Control is conceptualized in terms of organizational decision latitude, procedural justice, and conducive communication. Procedural justice refers to fairness in decision making; consistency of application; input from affected parties; and accurate, correctable, and ethical decisions [19]. Research on organizational justice showed that it was related to coronary heart disease (CHD; [20]). Specifically, low levels of procedural justice were associated with a higher risk of poor SRH and minor psychiatric disorders as well as sickness absence (women only), even after adjustment for the DCS task level measures (women only; [19]). Organizational decision latitude refers to control at the organizational level, provided by communication between employees and managers, which relates to subjective wellbeing and organizational commitment [21]. Conducive communication refers to a skill-enhancing, interactive communication mode engaging workers and customers.

Stability-support is conceived in terms of psychosocial safety climate, rewards, and consideration of workers’ interests. Psychosocial safety climate refers to organizational policies, practices, and procedures for the protection of worker psychological health and well-being and predicts psychological distress, emotional exhaustion, and employee engagement [22].

The inclusion of rewards, specifically the aspects of financial rewards and appreciation, is inspired by research on the effort–reward imbalance (ERI) model [23]. ERI is associated with depressive symptoms [24], adverse cardiovascular and other health effects [25], CVD and depression, Type II diabetes, alcoholism, sleeping problems, and sickness absence [26]. Both ERI and job strain are related to angina pectoris, depression, and poor SRH [27]. A systematic review found statistically significant associations between ERI and CVD [2].

Consideration of workers’ interests refers to fairness to workers in the context of organization change processes (see Appendix A, A.1).

We propose that the DCS-S constructs and their combinations (e.g., high strain, active work) at the organizational level will relate to health and work outcomes in parallel ways to the DCS constructs and combinations at the task level. We expect:

**Hypothesis** **5.** High organizational level demands will be positively associated with indicators of poor health and negatively associated with favorable work outcomes.

**Hypothesis** **6.** High organizational level control and stability-support will be negatively associated with poor health and positively associated with favorable work outcomes.

**Hypothesis** **7.** Workers with high organizational level job strain have higher levels of poor health than workers who have low strain.

**Hypothesis** **8.** Workers with high organizational level active work have higher levels of favorable work outcomes than those who have passive jobs.

Two separate but related tasks remain: The first is to show that the JCQ 2.0 tool can be used to verify theoretical considerations consistent with causal pathways—albeit only using cross-sectional data—that have important policy implications. The second task is more pragmatic and is to show the utility of using the JCQ 2.0 tool over the JCQ 1 tool.

Organizational context matters and affects work design [28], implying a hierarchy of causation. Therefore, one needs to consider how the organizational level DCS-S composites affect lower level entities, i.e., DCS at the task level. Since the organizational level is more distal, they are leading and likely to influence downstream task level composites. Demands emanating at the organizational level are likely to filter through to task level demands in addition to their direct effects on outcomes. Organizational level control is likely to affect task control, and stability-support at the organizational level is likely to affect task support, again both in addition to their direct effects on outcomes. For instance, psychosocial safety climate at the organizational level (work unit), reported by one group of nurses, predicted support from supervisors at the task level, reported by different nurses in the same unit 24 months later [29]. These hierarchical relations imply process paths from organizational DCS-S through task DCS to health and work outcomes. Hence, we propose:

**Hypothesis** **9.** Organizational composites (D, C, and S-S) will correlate with parallel task composites in such a way that organizational composites will affect task composites. Furthermore, task composites will affect health and work outcomes. There will be additional direct effects from organizational composites to health and work-related outcomes, not mediated by task composites.

Given these predicted multilevel associations, organizational composites will explain additional variance in health and work-related outcomes, as implied in the expectation of direct effects. The pragmatic goal here is to show instrument improvement, the added variance of JCQ 2.0 organizational domains over task domains, and vice versa. In addition, the composites at both levels assess working conditions in a far more comprehensive way than the JCQ 1 DCS core scales did. Hence, we aim to show the added variance of the new JCQ 2.0 tool over the established JCQ 1 DCS core, including the most often tested DC model measures: “psychological demands” and “decision latitude”. We expect:

**Hypothesis** **10.** JCQ 2.0 DCS-S composite scores at the organizational level will explain additional variance over and above that explained by the JCQ 2.0 composite scores at the task level and vice versa. Moreover, we expect that the JCQ 2.0 task and organizational composites will explain more variance than the JCQ 1 DC and DCS core scales.

## 2. Materials and Methods

### 2.1. Samples

Population-based samples were used in Australia and Germany. For detailed information about data collection across samples and a description of the samples, refer to Agbenyikey et al. (see Appendix A, A.2).

### 2.2. DCS-S Scales

The composite scales for the three dimensions D, C, and S-S at the task and organizational level comprise slightly differing items by country.

At the task level, the following scales have been used from the JCQ 1 in both Australia and Germany: quantitative demands (D), skill discretion and decision authority (C), and supervisor support and co-worker support (S). In both countries, emotional demands were added as a new scale for D. Moreover, the German questionnaire contained additional scales on conducive development (C) as well as collective control and negative acts (S).

At the organizational level, in both Australia and Germany, the scales restructuring (D), procedural justice and organizational decision latitude (C) as well as reward and psychosocial safety climate (S-S) were applied. In addition, the German questionnaire contained scales on organizational disorder (D), conducive communication (C), and consideration of workers’ interests (S-S).

### 2.3. Outcome Measures

We used six outcomes in the Australian and German pilot, three referring to health and three referring to work-related outcomes. For SRH, a single item from SF-36 was used [30], with a high score indicating poor self-rated health. Burnout was assessed with five items from the Maslach Burnout Inventory to assess emotional exhaustion [31] in Australia and with the seven-item scale “work-related burnout” from the Copenhagen Burnout Inventory [32] in Germany. In Australia, the complete PHQ-9 [33] was applied for assessing depression; in Germany, the scale’s first two items were used. High scores imply high levels of burnout and depression.

For the assessment of JS, a single item was used in Australia [34], and the seven-item scale from the Copenhagen Psychosocial Questionnaire [35] was used in Germany. Job commitment was assessed with the nine-item version of the Utrecht Work Engagement Scale [36] in Australia and with four items from the scale of affective commitment to the organization [37,38] in Germany. In Australia, ITS was assessed with a single item [39], while in Germany, workers were asked about their ITL [40]. All measures were kept as they were in the surveys; note this has implications for the direction of relationships. All outcome scale scores were computed as mean scores over the available items per scale.

### 2.4. Data Collection, Translation Process, and Data Handling

The data collection strategy in Australia and Germany, the translation process, the pre-testing, and the handling of missing data are described in detail in Agbenyikey et al. (see Appendix A, A.2).

### 2.5. Demand, Control, and Stability-Support Score Construction

For each scale included in the JCQ 2.0, the scale score was computed as the mean score over all items belonging to this scale. Cronbach’s alpha of these scales is presented in Agbenyikey et al. (see Appendix A, A.2). D, C, and S-S at the task and organizational level (where available) were computed by z-standardizing these JCQ 2.0 scale scores and combining scores belonging to each factor. Composite D, C, and S-S scores were used for both analyses of variance and path analyses. The JCQ 1 D, C, and S scores were calculated as weighted sum scores according to the JCQ 1.7 User’s Guide [15].

### 2.6. Construction of Job Strain and Active Work Scores

Job strain and active work were formulated on the basis of the composite D and C scores using the five-category version of the DC model [41] leading to five distinct groups—low strain, high strain, passive work, active work, and mid-population.

### 2.7. Correlation Analyses—D, C, and S-S with Outcomes

For correlation analyses between D, C, and S-S and the outcomes, Spearman’s correlation was used in order to account for the categorical nature of several outcomes. A value of |*r*| ≥ 0.1 implies a small effect, |*r*| ≥ 0.3 a medium effect, and |*r*| ≥ 0.5 a large effect [42]. Correlation analyses were based on the 10 imputed datasets; for information on handling missing data and multiple imputations, see Section 2.4 and Appendix A, A.2.

### 2.8. Comparisons of Outcomes for the Five Groups

Outcome scale scores (depression, burnout, JS, and AC in Germany, WE in Australia) for the five groups—low strain, high strain, passive work, active work, and mid-population—were compared using ANOVA and the Tamhane test for post-hoc comparisons to account for inequality of variances. For single-item outcome scores (SRH, ITL/ITS, JS in Australia), the Kruskal–Wallis test, which accounts for the ordinal nature, was used. The Mann–Whitney test was applied for post-hoc pairwise comparisons, requiring correction of the alpha level manually because of 10 pairwise tests, implying that only tests with α < 0.005 were considered statistically significantly different. For each pairwise comparison, Cohen’s *d* was calculated as a standardized measure of effect based on differences between means by applying the formula $d=\frac{x_{1}-x_{2}}{s}$ with *x*_1_ and *x*_2_ the mean scores for the two groups and *s* as the pooled variance, with |*d*| ≥ 0.2 indicating a small effect, |*d*| ≥ 0.5 a medium effect, and |*d*| ≥ 0.8 a large effect [42]. This approach was followed because with the large sample sizes in both Australia and Germany, very small differences are statistically significant, indicating that it is hence much more relevant to focus on effect sizes instead. Mean score comparisons were based on the 10 imputed datasets; see Section 2.4 and Appendix A, A.2.

### 2.9. Path Analysis

We used the composite DCS-S measures to test sequential path models. In two separate models, we regressed burnout and depression, respectively, on the task level composite and the organizational level composite and allowed for an additional path from the organizational level composite to the task level composite. These analyses were conducted separately for the domains demand, control, and stability-support. Burnout and depression were chosen as outcomes in our analyses because they are strongly associated with the DCS-S model according to empirical evidence over the past decades [6,43].

### 2.10. Added Variance

Using regression analysis, we determined the adjusted explained variance *R_adj_^2^* in incremental models, commencing with gender and age, adding JCQ 2.0 task demand, control, and support, then JCQ 2.0 organizational demand, control, and stability-support. In a second model, after controlling for gender and age, JCQ 2.0 organizational demand, control, and stability-support were added, followed by JCQ 2.0 task demand, control, and support. In a third and fourth model, the JCQ 1 DC and JCQ 1 DCS scores, respectively, for demand, control, and support were used as predictors after controlling for gender and age.

## 3. Results

### 3.1. Task Level

Hypothesis 1 proposed that high task demands will be positively associated with indicators of poor health (poor SRH, high levels of burnout, and depression) and negatively associated with favorable work outcomes (AC/WE, JS, and ITL/ITS). This hypothesis was nearly fully supported in Australia (no effect for demand and WE) and fully supported in Germany, as shown in Table 1; effects were of small to medium magnitude in most instances and large for burnout in Germany.

Hypothesis 2 proposed that high task control and support will be negatively associated with indicators of poor health and positively associated with favorable work outcomes. This hypothesis was nearly fully supported—as shown in Table 1—in Australia and Germany (no effect for control and burnout in Australia). For Australia, the effects were small to medium. In Germany, effects ranged from small to large.

### 3.2. Task-Level Job Combinations—Job Strain and Active Work

As shown in Table 2, for both Australia and Germany, Hypothesis 3—workers with high task-level job strain have higher levels of poor SRH, burnout, and depression compared to low-strain workers—was supported with small to large effects in Australia and large effects in Germany. Health among workers in high-strain jobs was significantly worse compared to those in low-strain jobs.

For Hypothesis 4—workers with high task-level active work have more favorable work outcomes than workers who have passive jobs—there was only support from the Australian data in relation to one out of three outcome measures, with a moderate effect for WE (Table 2). The German sample showed a negligible effect for ITL, a small effect for JS, and a moderate effect for AC (see Table 2).

### 3.3. Organizational Level

Hypothesis 5 proposed that high organizational demands are positively associated with indicators of poor health and negatively associated with favorable work outcomes. This hypothesis was supported in Germany and only partly supported in Australia, particularly in relation to favorable work outcomes (see Table 1). Organizational demands were related to health and work outcomes in Australia with small effects for burnout, depression, and JS (for poor SRH, WE, and ITS, the effect was negligible) and as expected, in Germany with small to moderate effects, with a large effect for JS.

Hypothesis 6 proposed that high organizational control and stability-support are negatively associated with indicators of poor health and positively associated with favorable work outcomes. Organizational-level control and stability-support were related to health and work outcomes as expected with small to medium effects in Australia and with small to large effects in Germany (see Table 1). Thus, this hypothesis was well supported.

### 3.4. Organizational-Level Job Combinations—Job Strain and Active Work

In Hypothesis 7, it was expected that workers with high organizational-level job strain have higher levels of poor health than workers who have low strain. This hypothesis was fully supported in Australia and Germany (see Table 2). In Australia, the effect was small for SRH and depression and large for burnout; for Germany, the results were even stronger (mainly large effects). In each case, workers’ health in organizational-level high-strain jobs was significantly worse compared to workers’ health in low-strain jobs.

Hypothesis 8 proposed that workers with high active work at the organizational level have higher levels of favorable work outcomes than those in passive work. This hypothesis was supported in Australia and Germany with small to moderate effects (see Table 2).

### 3.5. Path Analysis

**Figure 1:**
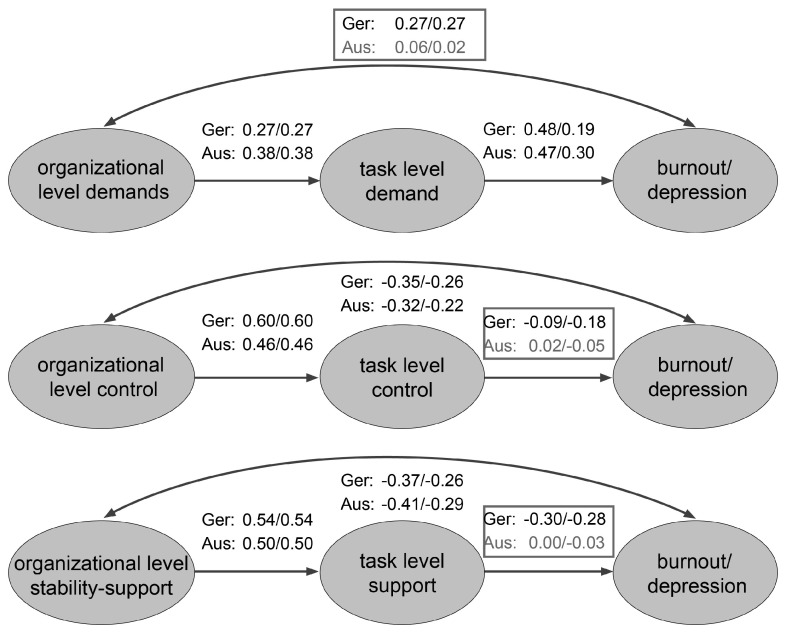
Path model—organizational level, task level, and burnout (before the slash) and depression (after the slash) in Australia and Germany. Values are standardized path coefficients β. “org” = organizational. Ger = Germany, Aus = Australia.

In the path models, organizational composites (D, C, and S-S) were modeled as antecedents to parallel task composites (see Figure 1). Effect sizes were small to large, linking the parallel composites across Australian and German data.

For both burnout and depression, the direct association between organizational demands and outcomes was close to zero in Australia and small in Germany when all other relations were modeled. The association between task control and task support and the outcomes were close to zero in Australia when all other relations were modeled simultaneously. This was not the case in Germany with the exception of task control and burnout; in all other cases, the associations in the German sample were of a small magnitude.

In Germany, organizational control was directly related to both burnout and depression in a stronger way than task control, whereas associations for task and organizational stability-support were of approximately equal size. For burnout, the relation was stronger for task demand, whereas for depression, it was stronger for organizational demands. In both Germany and Australia, organizational and task control and stability-support were each more strongly related than organizational and task demand.

Because the models are saturated, model fit indices are not available, and model fit could not be assessed. Furthermore, modeling a correlation between the task and the organizational factors and considering both as predictors of burnout or depression, respectively, would give the same values as reported above. However, due to the cross-sectional nature of the data, it is not possible to assess the direction of effects.

Hypothesis 9 that organizational D, C, and S-S affect task composites and task composites affect psychological health, allowing for direct effects of organizational composites on burnout and depression, was nearly fully supported in the German data across all factors. For Australia, this model was not clearly evident: in the demand domain, no association between organizational demand and outcomes remained when task demand was modeled too, which implies a process whereby organizational demands relate to burnout and depression through task demands. By contrast, for control and support, the task composites were not related to the outcomes when the relations with organizational composites were modeled simultaneously.

### 3.6. Value Added

Hypothesis 10 proposed that the JCQ 2.0 DCS-S organizational composites would explain additional variance over and above that explained by the JCQ 2.0 task composites and vice versa after controlling for gender and age. In addition, the hypothesis proposed that JCQ 2.0 task and organizational domains would explain more variance than the classic JCQ 1 DC and DCS.

For Australia, task and organizational composites added unique variance to each other in the prediction of health and work measures after controlling for gender and age, except for SRH (see Table 3 first half).

Compared to the baseline classic JCQ 1 DC scales and the JCQ 1 DCS scales, the complete JCQ 2.0 explained more variance in all outcomes (final lines in Table 3). This indicates merit in the JCQ 2.0 measures over and above those of JCQ 1. Considering both the JCQ 2.0 task and organizational DCS-S scores together explains the largest amount of variance in outcomes.

For Germany, the results were similar and of a larger magnitude across all outcomes (see Table 4). Adding the JCQ 2.0 organizational DCS-S composites in addition to the JCQ 2.0 task DCS composites led to a rise in explained variance. In the same vein, JCQ 2.0 task DCS composites explained additional variance in all outcomes over and above JCQ 2.0 organizational composites. Furthermore, the JCQ 2.0 task and organizational DCS-S composites predicted a larger amount of variance in all outcome measures than the JCQ 1 DC and JCQ 1 DCS scales. Consequently, utilizing the JCQ 2.0 task and organizational DCS-S composites allowed for the greatest explanation of outcome scores.

When considering the relative improvement in explained variance in relation to the classic JCQ 1 psychological demand and decision latitude scales which have been used in many research papers, this improvement varied between 63% for SRH (from *R_adj_*^2^ = 0.08 to *R_adj_*^2^ = 0.13) and 153% for AC (from *R_adj_*^2^ = 0.13 to *R_adj_*^2^ = 0.33) in Germany and between 33% for SRH (from *R_adj_*^2^ = 0.03 to *R_adj_*^2^ = 0.04) and 88% for JS (from *R_adj_*^2^ = 0.17 to *R_adj_*^2^ = 0.32) in Australia. In relation to the JCQ 1 DCS scales, the relative improvement varied between 26% for JS (from *R_adj_*^2^ = 0.50 to *R_adj_*^2^ = 0.63) and 47% for depression (from *R_adj_*^2^ = 0.17 to *R_adj_*^2^ = 0.25) in Germany and between 33% for SRH (from *R_adj_*^2^ = 0.03 to *R_adj_*^2^ = 0.04) as well as ITS (from *R_adj_*^2^ = 0.09 to *R_adj_*^2^ = 0.12) and 60% for JS (from *R_adj_*^2^ = 0.20 to *R_adj_*^2^ = 0.32) in Australia.

To summarize, Hypothesis 10 was supported since in both countries, the JCQ 2.0 task and organizational DCS-S composites explained unique variance in all outcomes, and the JCQ 2.0 DCS version explained more variance in all outcomes compared to the classic JCQ 1 DC and DCS versions.

### 3.7. Summary of Results

Table 5 summarizes the results of all hypotheses tests. The first three hypotheses were also tested in Korea and China; the details of these analyses are beyond the scope of this paper.

## 4. Discussion

In this paper, we were able to show that JCQ 2.0 task demand, control, and support relate to health and to positive work-related outcomes nearly as expected in Australia and as expected in Germany, nearly and fully confirming Hypotheses 1 and 2. These DCS JCQ 2.0 task composites comprise both the established JCQ 1 core scales as well as newly developed scales which capture working conditions at the task level in a more comprehensive way. Importantly, the meaning of the foundational concepts of demand and control was retained in the revision of the JCQ. In the same vein, task-level job strain relates to health and work-related outcomes in expected ways, confirming Hypothesis 3 and yielding further support for the claim that the revision of the JCQ has not undermined foundational concepts.

The JCQ 2.0 organizational-level scores for demand, control, and stability-support show a pattern of associations with outcomes that is in most cases similar to the one at the task level, thus fully confirming Hypothesis 6 in both samples and Hypotheses 5 in the German sample. In Australia, Hypothesis 5 could only partly be confirmed as there were some non-significant associations in the Australian sample in relation to organizational demands. In addition, associations between organizational-level job strain and health and work-related outcomes are similar to those for task-level strain, confirming Hypothesis 7 and implying similar mechanisms underlying the detrimental effect of poor working conditions at both the task and the organizational level on health.

By contrast, it was not possible to unequivocally confirm Hypothesis 4 regarding the association of task-level active work with positive work-related outcomes. There was only one association in the Australian data with WE; associations in the German data were present for two out of three outcomes (AC and JS). The argument that job satisfaction and job commitment may not be the most appropriate for testing the active work construct [44] does not hold true in the current study: in both countries, there was support for the association of active work and WE/AC; additionally, the relation to JS was present in the German sample. One possible explanation for the discrepancy between the two countries with regard to JS is that it was only measured with a single item in Australia and with a seven-item scale in Germany. However, since task demand, control, and support were related to JS in the Australian sample, this does not seem to be the reason. The above argument [44] could be applicable to ITS/ITL as an outcome measure. Another reason could be that we used the five-category version of the DC model [41], whereas a lot of other studies have used the four-category version as originally presented [11].

The active work hypothesis has been confirmed previously when additional outcomes were considered. A study with over 3000 employees in 76 northern Belgium companies provided support for the active work hypothesis with a new measure of innovative work behavior that comprises behaviors such as “finding original solutions” and “developing innovative ideas” [45]. This measure was also positively associated with work demands (i.e., work pressure), as hypothesized for demands in the DC model [11] and in the concept of “flow” according to Csikszentmihalyi [46]. The active work hypothesis has also been confirmed in relation to outside-work behaviors [12] and post-retirement social engagement [47].

In addition, it must be noted that practitioners have used the active work construct for observing real behavioral change. Social policy initiatives like the European Workplace Innovation Network EUWIN (https://workplaceinnovation.eu/this-is-workplace-innovation/, accessed on 13 March 2025) consider the active work construct, as do intervention projects like the “Tzatziki Work Organization Simulation Game” (http://www.oresundsynergy.com/tzatziki-game/, accessed on 13 March 2025). Much of this research has been “case study-based” in dynamically changing company environments, with the result of little direct questionnaire research and limited quantitative precision [48].

Nevertheless, further explanations seem to exist for the only partially supporting results regarding Hypothesis 4 because Hypothesis 8 on the association of organizational-level active work and positive work-related outcomes was fully confirmed, supporting the theoretical meaning of the active work hypothesis at the organizational level. Thus, the diverging results might be attributable to differences in organizational culture and/or occupational regulations and/or national labor policies, and/or their implementation between Germany and Australia at the time of the study that stimulate varying strategies at the organizational level. Consequently, these results call for further research on the active work hypothesis at both the task and the organizational level in relation to a wide range of outcomes that relate to learning behavior as proposed in the DC model [11], for which the JCQ 2.0 could prove to be a valuable tool.

For Hypothesis 9, results differed between Germany and Australia. The German data nearly fully confirmed the hypothesis that there are both direct as well as indirect effects of the organizational composites demand, control, and stability-support on burnout and depression, with the indirect effects modeled by the intermediate task composites. However, in Australia, no such evidence could be found, and either the direct effects (for demands) or the indirect effects (for control and stability-support) were zero or close to zero. It is possible that these diverging results are due to the fact that fewer scales were available in the Australian data compared to the German data, rendering the composite demand, control, and stability-support scores at both levels less comprehensive and stable in the Australian data. It is hence worthwhile to put this hypothesis to another test in Australia, applying the complete JCQ 2.0 with all researcher scales. Nevertheless, the direct effects of the organizational control and support measures in Australia show their importance above task-level effects and justify their measurement. The differences could also reflect a cultural difference whereby Australian workers find support and control from the top to be most comforting for their health and wellbeing.

In relation to demands, for both countries, results were consistent with the multilevel theorization that organizational demands are distal to psychological health (burnout and depression), and their detrimental effects on psychological health operate via their influence on task demands. This multilevel process was also observed in Germany for stability-support and control (for the latter, only for depression). In sum, in seven out of twelve tests, there was support for a multilevel mediation process whereby the more distal organizational composites were related to proximal task composites that affected burnout and depression.

Both task and organizational composites explained unique variance in accordance with Hypothesis 10, confirming that they are important for both health and work-related outcomes. With the exception of JS in Australia and AC in Germany, for all other outcomes, the unique variance explained by the task composites was slightly higher than the unique variance explained by the organizational composites. One likely explanation is the greater proximity of the task-level characteristics for workers.

Table 3 and Table 4 final regressions showed that there is substantial improvement in variance explained (indicated via *R_adj_*^2^) for each dependent variable with the use of the full JCQ 2.0 DCS-S composite scales with their broad theoretical coverage compared to the JCQ 1 DC and DCS scores. Further additional variance could possibly be explained when considering the JCQ 2.0 external-to-work scales (see Appendix A, A.5). The absolute magnitude of baseline and increase varied widely because the outcome measures differed in how proximal vs. distal they are to the work environment: whereas JS is clearly work-related, SRH is influenced by many other factors than solely the work environment.

Consistent with results of path analyses, the associations in the German data were of a larger magnitude than associations in the Australian data, possibly because the higher number of JCQ 2.0 scales available in the German data rendered the composite scores more stable. The full ADC theory’s relevant set of scales with substantial extensions in the three areas of demand, control, and stability-support was present only in the German scale set. Together with the greater clarity of the multilevel path model testing in Germany in comparison to Australia, the case is also made for the explanation that the full ADC theory, with its relevant dimensions, must be assessed by the JCQ 2.0 for it to reach both its full explanatory power and its ability to distinguish appropriately between the task and the organizational level. Further research is needed to confirm this latter interpretation.

Summarizing, Hypothesis 10 is confirmed: substantially more variance is explained in almost all outcome measures due to the JCQ 2.0 compared to the classic JCQ 1 DC and DCS core scales, which underlines the ability of the revised JCQ 2.0 to provide significantly more precise prediction of relevant outcomes compared to the earlier instrument.

### Strengths and Limitations of the Study

There are several strengths of the paper. The first is that the research hypotheses were largely confirmed in two different countries, Germany and Australia, with different cultures. Moreover, the study samples were large and from multiple occupations, allowing us to cover a wide spectrum of working conditions. Also, the revised JCQ 2.0 used in the study was systematically developed, allowing for a stepwise inclusion of more scales from the first study in Korea to the final study in Germany for greater domain coverage. Revisions were based on theoretical considerations as well as experiences in practical use, rendering the JCQ 2.0 suitable for use in practical contexts. Further, the outcome measures applied in the study in many cases stemmed from validated and established instruments. Finally, multiple imputation was applied to missing data instead of restricting analyses to complete cases, thereby avoiding over- or underestimation. As is described by Formazin et al. (see Appendix A, A.4) with regard to the German data, results from complete case analyses would have led to higher estimates, i.e., overestimation, compared to analyses with imputed data.

There are some limitations that need to be mentioned. This study is based on cross-sectional data; hence, it is not possible to address temporal association or causal interpretation of associations. It should be noted that our aim was rather to demonstrate the utility of the revised and enhanced JCQ 2.0 by illustrating how well it relates to health and work-related outcomes in comparison to established associations with the original JCQ 1. While the ADC model attempts to provide hypotheses about how changes in psychosocial working conditions could affect health and wellbeing outcomes, the restriction to cross-sectional data in this paper makes it impossible to fully test more dynamic aspects of the ADC hypotheses presented. Certainly, dynamic testing of the full ADC model is thus a necessary empirical next step for the future.

The German sample considered in the study was compared with available register information on the working population living in the city where the study took place (“Mikrozensus”; https://www.destatis.de/DE/ZahlenFakten/GesellschaftStaat/Bevoelkerung/Mikrozensus.html, accessed on 15 January 2013), indicating lower response among three groups of people: male workers, younger employees, and those with lower levels of education. At the same time, response was higher among the following: those older than 45, women, and those with a higher education level (see also Appendix A, A.4). As a limitation, one hence has to state that associations might be elevated due to unevenly distributed non-response. As a strength, participants stemmed from very different occupations.

McLinton [49] assessed the demographics in the randomly selected Australian data against Australian Bureau of Statistics data and found the data to be representative of the national working population sample and job characteristics, supporting the generalizability of the findings.

Since both working conditions and outcomes were self-reported, a common method explanation of the relationships cannot be ruled out. It can hence not be ruled out that associations between working conditions and outcomes are somewhat inflated. However, due to the way the study was designed, it was not possible to assess the working conditions or the health and work-related outcomes via a different method, e.g., through experts.

The iterative JCQ 2.0 development process, allowing for additional scales and items at later stages, led to data in four countries that cannot be compared easily nor analyzed simultaneously for direct comparisons. This is especially true for organizational-level constructs assessed in a limited way in the first two pilots (Korea and China) and therefore not included in this paper. Furthermore, this prevented us from testing the predictive models in these two countries, which would have been an additional strength otherwise. In addition, different health and work-related outcomes were used in the four pilots, and no positive outcomes (e.g., WE, JS) were assessed in the first two pilots, rendering direct comparisons between countries impossible. Further, the measures applied were not the most appropriate ones to test the active work hypothesis (which was only partially confirmed). Therefore, we call for further tests applying more suitable measures that directly assess learning behavior as suggested by DC theory [11]. Finally, future longitudinal research should pay attention to operationalizing the concepts at the correct level, for instance, sampling data across organizations and assessing organizational concepts at the organizational level through aggregation.

## 5. Conclusions

The results underline the utility of the JCQ 2.0 to assess multilevel aspects of the psychosocial work environment with broad practical value as a psychosocial risk assessment tool.

## Figures and Tables

**Table 1:** Task- and organizational-level D, C, and S-S correlations with health and work outcomes.

Australia (*N* = 4214)	Self-Rated Health ^+^	Burnout	Depression	Work Engagement	Job Satisfaction	Intention to Stay
Task demand	0.11	0.48	0.33	−0.07	−0.27	−0.14
Task control	−0.11	−0.08	−0.11	0.36	0.31	0.23
Task support	−0.13	−0.18	−0.13	0.25	0.36	0.27
Organizational demand	0.08	0.24	0.16	−0.04	−0.17	−0.08
Organizational control	−0.13	−0.28	−0.23	0.33	0.41	0.25
Organizational stability-support	−0.16	−0.38	−0.30	0.32	0.49	0.30
**Germany** **(*N* = 2326)**	**Self-Rated Health ^+^**	**Burnout**	**Depression**	**Affective** **Commitment**	**Job Satisfaction**	**Intention to Leave**
Task demand	0.24	0.55	0.28	−0.14	−0.30	0.26
Task control	−0.26	−0.27	−0.33	0.39	0.52	−0.26
Task support	−0.33	−0.48	−0.42	0.44	0.67	−0.38
Organizational demand	0.23	0.40	0.34	−0.38	−0.51	0.31
Organizational control	−0.28	−0.39	−0.37	0.47	0.64	−0.36
Organizational stability-support	−0.31	−0.52	−0.40	0.48	0.63	−0.45

Composite demand, control, stability-support measures; Spearman’s correlation; ^+^ a high value indicates poor SRH. All correlations have *p* < 0.01.

**Table 2:** Five-quadrant model comparisons to test job strain and active–passive hypotheses.

Australia (*N* = 4214)	Self-Rated Health ^+^	Burnout	Depression	Work Engagement	Job Satisfaction	Intention to Stay
Task level						
Low strain vs. high strain	−0.46	−1.95	−0.38	0.85	1.51	0.91
Passive work vs. active work	−0.06	−1.18	−0.15	−0.52	−0.07	−0.11
Organizational level						
Low strain vs. high strain	−0.35	−1.54	−0.29	0.68	1.26	0.73
Passive work vs. active work	0.12	0.18	0.07	−0.63	−0.69	−0.48
**Germany** **(*N* = 2326)**	**Self-Rated Health ^+^**	**Burnout**	**Depression**	**Affective** **Commitment**	**Job** **Satisfaction**	**Intention to Leave**
Task level						
Low strain vs. high strain	−1.04	−2.01	−1.21	1.09	1.80	−0.92
Passive work vs. active work	0.06	−0.56	0.15	−0.53	−0.46	0.08
Organizational level						
Low strain vs. high strain	−0.77	−1.34	−1.11	1.36	1.98	−1.00
Passive work vs. active work	0.19	0.04	0.21	−0.46	−0.68	0.36

Composite demand, control measures; results are “*d*” statistics: standardized mean differences between selected quadrants; five-quadrant model: four quadrants plus a mid-population; ^+^ a high value indicates poor SRH.

**Table 3:** Regression of health and work outcomes on different JCQ formulations—Australian data.

Australia (*N* = 4214)	Self-Rated Health ^+^	Burnout	Depression	Work Engagement	Job Satisfaction	Intention to Stay
Total adjusted *R^2^*— Gender and age	0.00	0.02	0.02	0.01	0.003	0.008
Model 1—Adjusted *R^2^* change						
Step 1. JCQ 2.0 DCS task	0.04	0.27	0.12	0.14	0.24	0.10
Step 2. JCQ 2.0 DCS-S org	0.004	0.04	0.02	0.04	0.08	0.02
Model 2—Adjusted *R^2^* change						
Step 1. JCQ 2.0 DCS-S org	0.04	0.20	0.10	0.13	0.29	0.10
Step 2. JCQ 2.0 DCS task	0.004	0.11	0.04	0.05	0.04	0.02
Total adjusted *R^2^*— JCQ 2.0 DCS -S	0.04	0.31	0.14	0.18	0.32	0.12
Model 3—Total adjusted *R^2^* baseline JCQ 1 DC	0.03	0.21	0.09	0.12	0.17	0.07
Model 4—Total adjusted *R^2^* baseline JCQ 1 DCS	0.03	0.23	0.09	0.13	0.20	0.09
Difference: Complete JCQ 2.0 DCS-S—JCQ 1 DCS	0.01	0.08	0.05	0.05	0.12	0.03
Difference: Complete JCQ 2.0 DCS-S—JCQ 1 DC	0.01	0.10	0.05	0.06	0.15	0.05

Following line 1, results are corrected for the variance explained by gender and age. ^+^ A high value indicates poor SRH. Single values not adding up to the sum are due to rounding.

**Table 4:** Regression of health and work outcomes on different JCQ formulations—German data.

Germany (*N* = 2326)	Self-Rated Health ^+^	Burnout	Depression	Affective Commitment	Job Satisfaction	Intention to Leave
Total adjusted *R^2^*— Gender and age	0.08	0.002	0.002	0.00	0.00	0.05
Model 1—Adjusted *R^2^* change						
Step 1. JCQ 2.0 DCS task	0.12	0.44	0.23	0.25	0.55	0.22
Step 2. JCQ 2.0 DCS-S org	0.01	0.04	0.02	0.08	0.08	0.03
Model 2—Adjusted *R^2^* change						
Step 1. JCQ 2.0 DCS-S org	0.10	0.32	0.20	0.30	0.55	0.21
Step 2. JCQ 2.0 DCS task	0.03	0.16	0.05	0.03	0.09	0.04
Total adjusted *R^2^*— JCQ 2.0 DCS-S	0.13	0.48	0.25	0.33	0.63	0.25
Model 3—Total adjusted *R^2^* baseline JCQ 1 DC	0.08	0.28	0.13	0.13	0.27	0.11
Model 4—Total adjusted *R^2^* baseline JCQ 1 DCS	0.10	0.35	0.17	0.25	0.50	0.19
Difference: Complete JCQ 2.0 DCS-S—JCQ 1 DCS	0.04	0.13	0.08	0.08	0.14	0.06
Difference: Complete JCQ 2.0 DCS-S—JCQ 1 DC	0.05	0.20	0.12	0.20	0.36	0.14

Following line 1, results are corrected for the variance explained by gender and age. ^+^ A high value indicates poor SRH. Single values not adding up to the sum are due to rounding.

**Table 5:** Overview of hypotheses confirmation across four countries.

With Health and/or Work Outcomes	Korea	China	Australia	Germany
H1. Task D with all	partly	confirmed	nearly fully confirmed	confirmed
H2. Task C and S with all	partly	confirmed	nearly fully confirmed	confirmed
H3. Task strain with health	confirmed	confirmed	confirmed	confirmed
H4. Task active with work-related outcomes	-	-	partly (only 1 out of 3)	partly (2 out of 3)
H5. Org D with all	-	-	partly	confirmed
H6. Org C and S-S with all	-	-	confirmed	confirmed
H7. Org strain with health	-	-	confirmed	confirmed
H8. Org active with work-related outcomes	-	-	confirmed	confirmed
H9. Task and org with direct and indirect effects	-	-	not	nearly fully confirmed
H10. Added variance	-	-	confirmed	confirmed

H: hypothesis; Org: organizational; D: demand; C: control; S: support; S-S: stability-support.

## Data Availability

Due to data privacy regulations, the data from Germany, Korea, and China cannot be distributed. Australian Workplace Barometer Data is available at the Australian Data Archive, Australian National University.

## Appendix A. References to Papers in the Special Issue “The Job Content Questionnaire 2.0: A Tool for Measurement of the Psychosocial Work Environment and Sustainable Work Globally”

**A.1** Karasek, R.; Dollard, M.F.; Östergren, P.-O.; Cho, S.-i.; Houtman, I. The multi-level Job Content Questionnaire 2.0 (JCQ 2.0) and the Associationalist Demand–Control (ADC) Theory for a sustainable global economy. *Int. J. Environ. Res. Public Health* **submitted**.

**A.2** Agbenyikey, W.; Li, J.; Cho, S.-i.; McLinton, S.S.; Dollard, M.F.; Formazin, M.; Choi, B.; Houtman, I.; Karasek, R. International comparative reliability and concurrent validity assessment of the multi-level Job Content Questionnaire (JCQ) 2.0. *Int. J. Environ. Res. Public Health* **submitted**.

**A.3** Formazin, M.; Choi, B.; Dollard, M.F.; Li, J.; McLinton, S.S.; Agbenyikey, W.; Cho, S.-i.; Houtman, I.; Karasek, R. The structure of demand, control, and stability-support underlying the Job Content Questionnaire (JCQ) 2.0. *Int. J. Environ. Res. Public Health* **submitted**.

**A.4** Formazin, M.; Martus, P.; Burr, H.; Pohrt, A.; Choi, B.; Karasek, R. The cross-sectional association of scales from the Job Content Questionnaire 2 (JCQ 2.0) with burnout and affective commitment among German employees. *Int. J. Environ. Res. Public Health* **2025**, *22*, 386. https://doi.org/10.3390/ijerph22030386.

**A.5** Agbenyikey, W.; Karasek, R.; Formazin, M.; Cho, S.-i.; Choi, B.; Dollard, M.F.; Li, J.; Houtman, I. Associations of external-to-work scales of the Job Content Questionnaire (JCQ) 2.0 with relevant outcome measures. *Int. J. Environ. Res. Public Health* **submitted**.
